# High dupilumab levels in tear fluid of atopic dermatitis patients with moderate‐to‐severe ocular surface disease

**DOI:** 10.1002/clt2.12221

**Published:** 2023-01-16

**Authors:** Roselie Achten, Judith Thijs, Marlot van der Wal, Chantal van Luijk, Matthijs van Luin, Mohsin el Amrani, Edward Knol, Eveline Delemarre, Constance den Hartog Jager, Marlies de Graaf, Daphne Bakker, Joke de Boer, Femke van Wijk, Marjolein de Bruin‐Weller

**Affiliations:** ^1^ Department of Dermatology and Allergology National Expertise Center for Atopic Dermatitis, University Medical Center Utrecht Utrecht The Netherlands; ^2^ Center for Translational Immunology University Medical Center Utrecht Utrecht University Utrecht The Netherlands; ^3^ Department of Ophthalmology University Medical Center Utrecht Utrecht The Netherlands; ^4^ Department of Clinical Pharmacy University Medical Center Utrecht Utrecht The Netherlands

**Keywords:** atopic dermatitis, dupilumab, ocular surface disease, tear fluid

## Abstract

**Background:**

The patho‐mechanism of ocular surface disease (OSD) in dupilumab‐treated atopic dermatitis (AD) patients remains unclear. The aim of this study is to measure dupilumab levels in tear fluid and serum, and relate these findings to the severity of OSD during dupilumab treatment in AD patients.

**Methods:**

This prospective study included dupilumab‐treated moderate‐to‐severe AD patients who were seen by a dermatologist and an ophthalmologist before the start of dupilumab (baseline), and after 4 and 28 weeks of dupilumab treatment. Dupilumab levels in tear fluid and serum were measured by liquid chromatography coupled with tandem mass spectrometry (LC‐MS/MS). Additionally, a pilot study was conducted to measure dupilumab on conjunctival epithelial cells using flow cytometry and LC‐MS/MS.

**Results:**

At baseline, 89.6% (*n* = 43/48) of the patients had OSD, with 50.0% having moderate‐to‐severe OSD. After 28 weeks of dupilumab treatment, the median dupilumab tear fluid levels were 0.55 mg/L (IQR 0.35–1.31) and 0.29 mg/L (IQR 0.16–0.60) in patients with moderate‐to‐severe OSD and patients with no or mild OSD, respectively (*p* = 0.02). Dupilumab levels could be detected on conjunctival epithelial cells of 5 AD patients treated with dupilumab for 4 weeks.

**Conclusion:**

Patients with moderate‐to‐severe OSD had higher dupilumab tear fluid levels compared to patients with no or mild OSD, indicating that dupilumab reaches the ocular surface. Dupilumab was also detected in conjunctival cell suspensions and was found to directly bind CD45‐conjunctival epithelial cells. This suggests that AD‐induced changes of the conjunctival epithelium may play a role in the development of OSD as well as increased local drug availability.

## 1 INTRODUCTION

Atopic dermatitis (AD) is a chronic, inflammatory, itchy skin disease with a prevalence up to 10% in adults.^1^, ^2^ The first biologic therapy that has been introduced to treat moderate‐to‐severe AD is dupilumab. Dupilumab is a fully human monoclonal IgG4 antibody that is directed against the interleukin (IL)‐4 receptor‐alpha (IL‐4Rα) subunit, inhibiting the binding of IL‐4 and IL‐13.^3^ It has proven its effectiveness in both clinical trials and daily practice studies.^3^, ^4^, ^5^ The most frequently reported adverse event during dupilumab treatment in AD patients is dupilumab‐associated ocular surface disease (DAOSD), which has been reported in up to 34% of the patients.^4^, ^5^, ^6^ Remarkably, in dupilumab trials in other type‐2 inflammatory diseases, such as asthma and chronic rhinosinusitis with nasal polyposis, no increased rates of DAOSD were reported.^6^ Recently we found that 90% of the moderate‐to‐severe AD patients had clinical characteristics of ocular surface disease (OSD) before the start of dupilumab, suggesting that AD patients may have a predisposition to develop DAOSD.^7^ It might be possible that this pre‐existent OSD is aggravated during dupilumab treatment and is then diagnosed as DAOSD.

Several hypothesis are suggested to be responsible for the development of DAOSD, such as focal scarcity of conjunctival goblet cells (GCs). This might be a result of the IL‐13 blocking effect, leading to reduced GC hyperplasia.^6^, ^8^ It is also hypothesised that DAOSD incidence may decrease in patients with higher serum dupilumab concentrations, and that local under‐treatment of dupilumab in the eyes might play a role in the development of DAOSD.^6^, ^9^ At this moment, the exact patho‐mechanism of DAOSD remains unclear.

To the best of our knowledge, no data are available on dupilumab levels in tear fluid of dupilumab‐treated AD patients. The aim of this study was to measure dupilumab levels in tear fluid and serum, and relate this to the severity of OSD during dupilumab treatment in AD patients.

## 2 MATERIALS AND METHODS

### 2.1 Study design and patients

This prospective monocenter observational cohort study included adult patients with moderate‐to‐severe AD between February 2020 and September 2021 from the University Medical Center Utrecht (UMCU), the Netherlands. Included patients were not using systemic immunosuppressive therapies for at least 2 weeks prior to initiation of dupilumab treatment.

All patients were examined by a dermatologist and an ophthalmologist before the start of dupilumab (baseline), and after 4 and 28 weeks of dupilumab treatment. At baseline, a full ophthalmological examination was performed after which patients received a 600 mg loading dose of dupilumab, followed by 300 mg injections every other week. If patients developed symptoms of OSD and/or worsening of pre‐existing OSD during dupilumab treatment, an extra ophthalmological examination was performed. In some patients, the week 4 or week 28 visit corresponded with the occurrence of OSD symptoms reported by patients and/or worsening of pre‐existing OSD during dupilumab treatment. The ophthalmologist started OSD treatment if patients had signs and symptoms of OSD, which might have influenced the OSD severity during dupilumab treatment. Treatment options included tacrolimus skin ointment for the eyelids, eye drops (including lubricants, antihistaminic, and corticosteroids), or eye ointment (including lubricants and corticosteroids). The selected therapy depended on the severity of OSD. All patients provided written informed consent for this study that was approved by the Institutional Review Board of the UMCU.

## 3 DATA COLLECTION

### 3.1 Dermatological and ophthalmological examination (baseline and during dupilumab treatment)

Clinical and dermatological data included patient characteristics, severity of AD based on the Eczema Area and Severity Index (EASI) and the Investigator's Global Assessment (IGA), and the presence or absence of other atopic comorbidities. In addition, ophthalmologic examination was performed according to the standardized Utrecht Ophthalmic Inflammatory and Allergic disease (UTOPIA) score, focussing on the severity of the inflammation of the conjunctiva, both tarsal and bulbar, the eyelids, and the limbus.^10^ An overall severity classification of no (UTOPIA score 0), mild (UTOPIA score 1–4), moderate (UTOPIA score 5–8), or severe OSD (UTOPIA score ≥9) was reported per patient per visit. Severity of OSD was based on the eye with the highest severity within a patient. During ophthalmological examination, topical anaesthesia eye drops (0.4% oxybuprocaine hydrochloride) were dripped in both eyes. Tear production was measured by a Schirmer's test.^11^ Subsequently, Schirmer's strips were air‐dried for at least 1 h and stored at −80°C until further analyses.

### 3.2 Processing of the Schirmer's strips

Schirmer's strips were eluted to obtain tear fluid for further processing. The elution buffer included PBS, Tween20 0.50%, BSA 1%, and 1 EDTA‐free Protease Inhibitor Cocktail tablet (Sigma). Every Schirmer's strip was cut into small pieces and placed in one well of a Falcon plate. Next, 100 μL elution buffer was added per well, the plate was sealed and incubated over night at 4°C on a shaker (230 RPM). The next day, a quick spin down was done for 1 min (1500 RPM). A MaxiSorp plate was taped under the MultiScreen filter plate, and the supernatant was collected. A spin down at 2100 G was conducted during 5 min for two times. The eluted tear fluid from both eyes were combined into a new Falcon plate and the eluted tear fluid was stored at −80°C until further analyses.

### 3.3 Measurement of dupilumab levels in tear fluid and serum

Dupilumab concentrations in both tear fluid and serum samples were measured with liquid chromatography tandem mass spectrometry (LC‐MS/MS) according to our in‐house developed method,^12^ with the following minor modifications for tear fluid measurements. In short, 10 µL sample was pipetted in 1 ml 96‐well plate and 10 µL SIL IFX was added followed by 10 µL bovine serum and 70 µL TRIS (50 mM, pH 8, 0.5% OG). Then, 100 µL saturated ammonium sulfate was added to each sample followed by 1 min mixing at 1350 RPM. The 96 well plate was centrifuged at 4000 G for 5 min. After the supernatant was decanted the pellet was reduced, alkylated, digested and measured following our in‐house developed method.^12^

### 3.4 Measurement of dupilumab on conjunctival epithelial cells

A pilot study was conducted to investigate the effect of dupilumab tear fluid levels on conjunctival cells. Conjunctival impression cytology (CIC) was collected from 3 control AD patients (not treated with dupilumab) and from 5 AD patients treated with dupilumab for 4 weeks. CIC was obtained as described previously, and stored in 100 µL PBS (Sigma) at −80°C until further analysis.^7^ LC‐MS/MS was used to measure dupilumab in CIC suspensions.

Additionally, in 4 different AD patients that were being treated with dupilumab for 4 weeks, flow cytometry analysis of CIC (collected in PBS (Sigma) containing 0.05% (w/v) paraformaldehyde (Alfa Aesar)) was conducted. Within 3 weeks of sample collection, cells were extracted by gentle manual agitation using a 0.70 µM easystrainer (Greiner Bio‐One) for 2 min. Surface staining of CD45 AF700 and anti‐IgG4‐biotin, as a marker for dupilumab binding, was performed for 25 min at 4°C, followed by 25 min incubation of the second antibody streptavidin‐APC at 4°C. Surface staining of IL‐4Rα (CD124) PE was performed for 25 min at 37°C, using an optimization protocol after testing different temperatures. Data acquisition was performed on a FACS Fortessa flow cytometer (BD Biosciences) and data was analysed using FlowJo Software (Tree Star Inc.)

### 3.5 Statistical analysis

Patient characteristics were described using absolute numbers (N) and percentages for categorical variables and median with interquartile ranges (IQR) for non‐normally distributed continuous variables. Differences in dupilumab levels between no or mild OSD and moderate‐to‐severe OSD were calculated using the Mann–Whitney *U* test. Correlations between dupilumab levels in tear fluid and serum, and UTOPIA scores were described using Spearman's test. A *p*‐value of less than 0.05 was considered statistically significant. Statistical analyses were conducted with SPSS Statistics version 26.0.0.1 (IBM Corp. IBM SPSS Statistics for Windows). Figures were created by Prism (version 9.3.0 GraphPad Software).

## 4 RESULTS

### 4.1 Baseline results

#### 4.1.1 Patient characteristics

A total of 48 moderate‐to‐severe dupilumab‐treated AD patients were included (Table 1). Median baseline EASI score was 16.4 (IQR 10.9–21.8). Before start of dupilumab, mild, moderate, and severe OSD were reported in 19/48 (39.6%), 17/48 (35.4%), and 7/48 (14.6%) patients, respectively. Only 5/48 (10.4%) patients had no OSD at baseline.

**TABLE 1:** Patient characteristics at baseline

	Total cohort (*n* = 48)
Age (years), median (IQR)	38 (27–48)
Men, *n* (%)	24 (50.0)
Age of onset of AD, *n* (%)	
Childhood	44 (91.7)
Adolescence	2 (4.2)
Adult	2 (4.2)
History of self‐reported episodic acute allergic conjunctivitis, n (%)	39 (81.3)
Allergic asthma, *n* (%)	23 (47.9)
Allergic rhinitis, *n* (%)	36 (75.0)
Food allergy, *n* (%)	27 (56.3)
History of rosacea, *n* (%)	2 (4.2)
EASI score, median (IQR)	16.4 (10.9–21.8)
IGA score, median (IQR)	3 (3–4)
AD eyelid involvement in the past year, *n* (%)	32 (66.7)
AD facial involvement in the past year, *n* (%)	45 (93.8)
TARC (pg./ml), median (IQR)	1553 (802–2402)
Peripheral blood eosinophils (×10^9^/L), median (IQR)	0.25 (0.15–0.44)
Eosinophilia (≥0.45×10^9^/L), *n* (%)	11 (22.9)
Severity of OSD before the start of dupilumab,^a^ *n* (%)	
No OSD	5 (10.4)
Mild OSD	19 (39.6)
Moderate OSD	17 (35.4)
Severe OSD	7 (14.6)

Abbreviations: AD, atopic dermatitis; EASI, Eczema Area and Severity Index; IGA scale, Investigator's Global Assessment Scale; IQR, interquartile range; OSD, Ocular Surface Disease; TARC, thymus and activation‐regulated chemokine.

^a^ Severity of OSD is based on eye with the highest severity within a patient.

### 4.2 Dupilumab levels in tear fluid and serum at week 4 and week 28

**FIGURE 1:**
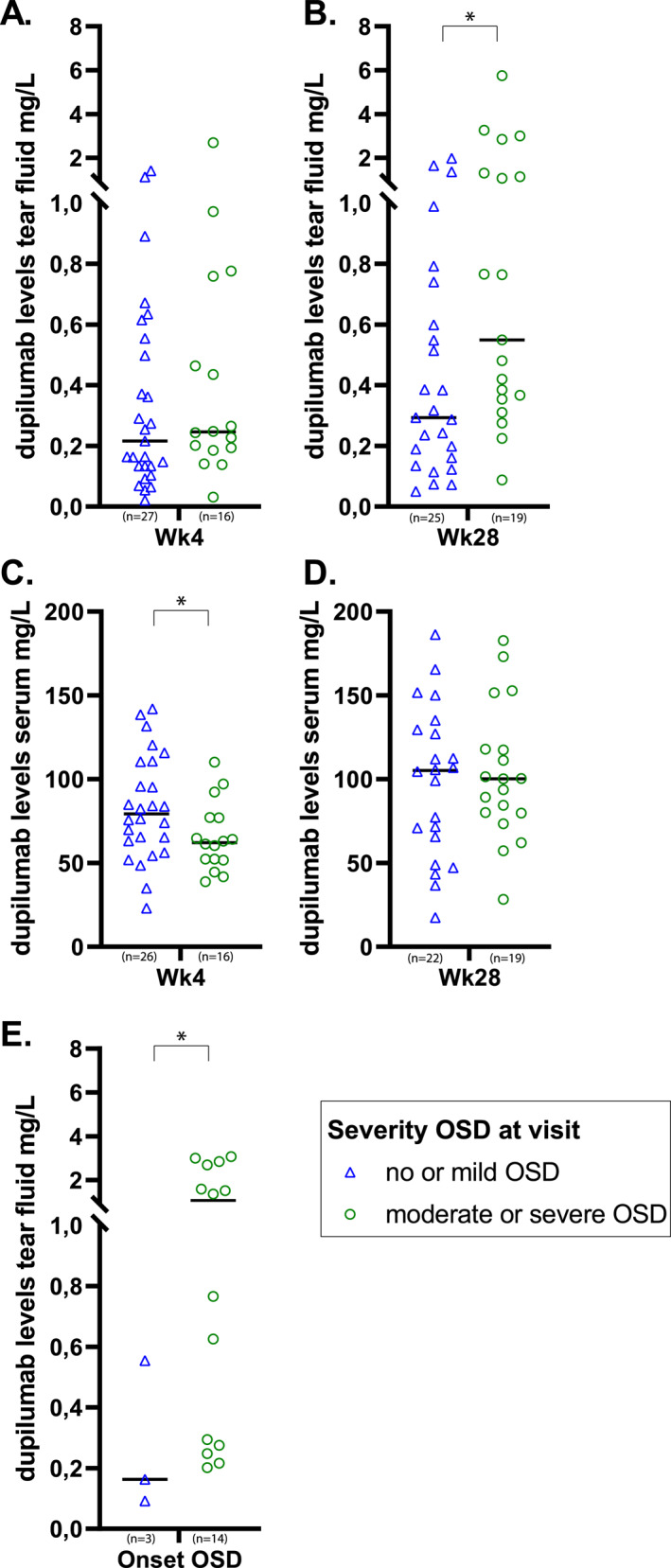
Dupilumab levels in tear fluid and serum. Severity of ocular surface disease (OSD) is based on the moment of tear fluid collection, and on the eye with the highest severity within a patient. Bold lines display the median. Differences were calculated with Mann‐Whitney U tests. (A), Tear fluid dupilumab at week 4 of dupilumab (*n* = 43). (B) Tear fluid dupilumab at week 28 of dupilumab (*n* = 44). (C) Serum dupilumab at week 4 of dupilumab (*n* = 42). (D) Serum dupilumab at week 28 of dupilumab (*n* = 41). (E) Tear fluid dupilumab at the additional ophthalmological examination which was scheduled due to development of OSD symptoms and/or worsening of ocular inflammation (onset OSD or worsening of pre‐existent OSD) (*n* = 17, 6 samples are missing). * Indicates statistical significance.

To investigate whether dupilumab is able to reach the ocular surface, we have measured dupilumab levels in tear fluid. At week 4 of dupilumab treatment, mild, moderate, and severe OSD were reported in 25/48 (52.1%), 12/48 (25.0%), and 4/48 (8.3%) patients, respectively. Data of 4 patients were missing due to COVID‐19. After 4 weeks of dupilumab treatment, patients with moderate‐to‐severe OSD at week 4 had comparable dupilumab tear fluid levels to patients with no or mild OSD at week 4 (0.25 mg/L (IQR 0.19–0.61) versus 0.22 mg/L (IQR 0.13–0.55), *p* = 0.35) (Figure 1A, Table S1). No correlation was found between dupilumab tear fluid levels at week 4 and UTOPIA score at week 4 of dupilumab treatment (Spearman's correlation 0.238, *p* = 0.124).

At week 28 of dupilumab treatment, mild, moderate, and severe OSD were reported in 25/48 (52.1%), 16/48 (33.3%), and 5/48 (10.4%) patients, respectively. After 28 weeks of dupilumab treatment, significant higher dupilumab tear fluid levels were found in patients with moderate‐to‐severe OSD at week 28 compared to patients with no or mild OSD at week 28 (0.55 mg/L (IQR 0.35–1.31) versus 0.29 mg/L (IQR 0.16–0.60), respectively, *p* = 0.02) (Figure 1B, Table S1). Additionally, a significant correlation between dupilumab tear fluid levels at week 28 and UTOPIA score at week 28 was found (Spearman's correlation 0.505, *p* < 0.001).

In contrast to dupilumab tear fluid levels, dupilumab serum levels after 4 weeks of dupilumab treatment were significantly lower in patients with moderate‐to‐severe OSD at week 4 compared to patients with no or mild OSD at week 4 (62.1 mg/L (IQR 52.0–77.0) versus 79.4 mg/L (IQR 63.2–110.5), *p* = 0.043) (Figure 1C, Table S1). After 28 weeks of treatment with dupilumab, no significant differences were found in dupilumab serum levels in patients with moderate‐to‐severe OSD at week 28 compared to patients with no or mild OSD at week 28 (Figure 1D, Table S1). Both at week 4 and week 28 of dupilumab treatment, no correlations were found between serum dupilumab levels and UTOPIA scores (Spearman's correlation −0.286, *p* = 0.066 at week 4 and Spearman's correlation 0.019, *p* = 0.907 at week 28).

### 4.3 Dupilumab tear fluid levels in patients with new onset OSD or worsening of pre‐existing OSD

Dupilumab levels in tear fluid at the onset of OSD (*n* = 3, 1 missing sample) or in case of worsening of pre‐existing OSD (*n* = 14, 5 missing samples) during dupilumab treatment were measured in 17 patients, samples of 6 patients were missing. Patients were divided into having mild OSD (*n* = 3, 2 missing samples) or moderate‐to‐severe OSD (*n* = 14, 4 missing samples). Significantly higher dupilumab levels were found in patients with moderate‐to‐severe OSD compared to patients with mild OSD (1.07 mg/L (IQR 0.28–2.69) vs. 0.16 mg/L (IQR 0.09–0.55), *p* = 0.047) (Figure 1E, Table S1).

Taken together this demonstrates that although dupilumab serum levels are lower (at week 4) or similar (at week 28) in patients with moderate‐to‐severe OSD compared to patients with no or mild OSD, local dupilumab levels in tear fluid do increase with increasing OSD severity.

### 4.4 Dupilumab on conjunctival cells

**FIGURE 2:**
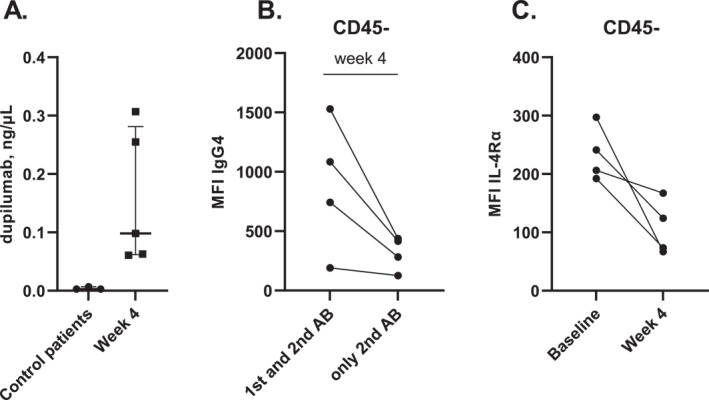
Dupilumab measured in conjunctival cells obtained by conjunctival impression cytology. (A) Dupilumab measured in conjunctival cell suspensions from 5 dupilumab‐treated AD patients (at week 4) and 3 AD control patients (not treated with dupilumab) by using LC‐MS/MS. (B) Median Fluorescence Intensity (MFI) of IgG4 (= anti‐dupilumab) in CD45‐epithelial cells from 4 dupilumab‐treated AD patients (at week 4) compared to the control staining including only the secondary antibody (AB) streptavidine‐APC. (C) MFI of IL‐4Rα on CD45‐cells from 4 AD patients before dupilumab treatment (baseline) and after 4 weeks of treatment.

The presence of dupilumab in tear fluid does not prove its binding and potential direct biological effect on conjunctival cells. Therefore we investigated dupilumab binding on conjunctival cells in a pilot study by analysing CIC suspensions with LC‐MS/MS and flow cytometry (Gating strategy in Figure S1, patient characteristics are shown in Table S2). Dupilumab levels could indeed be detected in the conjunctival cell suspensions of 5 AD patients treated with dupilumab for 4 weeks compared to 3 AD controls (Figure 2A).

Flow cytometry analysis confirmed IgG4 (dupilumab) binding on CD45‐epithelial cells in 4 AD patients treated with dupilumab for 4 weeks (Figure 2B). Furthermore, Median Fluorescence Intensity (MFI) of IL‐4Rα on CD45‐epithelial cells decreased after 4 weeks of dupilumab treatment compared to MFI of IL‐4Rα on CD45‐epithelial cells at baseline (Figure 2C). These data indicate that dupilumab present at the ocular surface may have a direct effect on the conjunctival epithelium by blocking IL‐4Rα.

## 5 DISCUSSION

This prospective study shows that OSD is very frequent in moderate‐to‐severe AD, and that dupilumab‐treated AD patients with moderate‐to‐severe OSD had higher dupilumab tear fluid levels compared to patients with no or mild OSD during dupilumab treatment. Additionally, dupilumab was detected in conjunctival cell suspensions of five AD patients after 4 weeks of treatment with dupilumab.

At week 4 of dupilumab treatment, lower serum dupilumab levels were found in patients with moderate‐to‐severe OSD. However, the steady‐state is achieved after 16 weeks of dupilumab treatment, which may explain the variation in the week 4 levels.^13^ After 28 weeks of dupilumab treatment, dupilumab serum levels of patients with no or mild OSD at week 28 were similar to dupilumab serum levels of patients with moderate‐to‐severe OSD at week 28, and no correlation was found between serum dupilumab levels and UTOPIA scores at week 28. However, tear fluid dupilumab levels were higher in patients with moderate‐to‐severe OSD at week 28 of dupilumab treatment. These findings are in contradiction with the hypothesis that development of DAOSD is related to local under‐treatment by dupilumab in the eyes, based on an inverse relationship between serum dupilumab levels and conjunctivitis.^9^ Furthermore, the hypothesis of Akinlade et al.,^6^ who suggested that conjunctivitis incidence might decrease with higher dupilumab concentrations is also not in line with our findings. Based on our results, higher serum levels of dupilumab do not prevent development of OSD. This is also supported by our previous findings that prolongation of the dosing interval of dupilumab or discontinuation of dupilumab resulted in improvement of DAOSD.^10^ In addition, we recently investigated dupilumab serum levels in moderate‐to‐severe AD patients after 16 weeks of treatment, and did not find an association between development of DAOSD and serum dupilumab levels.^14^ However, our current study indicates that higher levels of dupilumab in tear fluid might be related to the severity of OSD during dupilumab treatment, and UTOPIA scores at week 28 significantly correlated with dupilumab tear fluid levels at week 28. It is well possible that pre‐existent OSD increases local barrier permeability and dupilumab availability. This is supported by observations by Sebbag et al.^15^ who investigated the impact of mild or severe conjunctivitis on lacrimal drug levels of oral prednisolone in six dogs. They reported a larger amount of prednisolone in eyes with conjunctivitis compared to control eyes, and found significantly higher levels of prednisolone in eyes of dogs with severe conjunctivitis compared to mild conjunctivitis. Sebagg et al.^15^ suggested that increased permeability of conjunctival vessels due to conjunctivitis might lead to leakage of plasma constituents into the tear component, which is called the blood‐tear barrier. Yokoi et al.^16^ investigated the barrier function of the ocular surface epithelium in AD patients, and demonstrated impaired epithelium barrier function in the eyes of patients with AD and blepharoconjunctivitis. These findings suggest that AD patients with moderate‐to‐severe OSD might have a disrupted blood‐tear barrier, leading to significantly higher dupilumab tear fluid levels during treatment compared to patients with no or mild OSD. This leads to the question whether the increased dupilumab tear fluid levels affect local epithelial cell homeostasis and/or development.

We could detect dupilumab in conjunctival cell suspensions of dupilumab‐treated AD patients after 4 weeks of treatment with dupilumab. Additional analysis showed IgG4 binding, indicative of dupilumab binding since dupilumab was also measured in the conjunctival cell suspensions, on CD45‐epithelial cells after 4 weeks of treatment. The decrease in IL‐4Rα staining, which is the receptor targeted by dupilumab, on CD45‐cells after 4 weeks of dupilumab treatment indicates direct binding of dupilumab on the IL‐4Rα of conjunctival cells. The question is whether this has any biological and/or clinical consequences. A previous study by Ueta et al. showed that functional IL‐4Rα is expressed on human conjunctival epithelium.^17^ Recently, Hansen et al.^18^ demonstrated that human GCs also express IL‐4Rα and that both IL‐4 and IL‐13 are important for their homeostasis. Previously, we demonstrated scarcity of conjunctival GCs in patients who developed DAOSD.^8^ Based on our current data we hypothesize that dupilumab‐induced changes of conjunctival epithelium development may play a role in the development of OSD during dupilumab treatment, and that local dupilumab levels could have a worsening effect on DAOSD by interfering with GC development. Further research is needed to study the implications of the binding of dupilumab on the conjunctival epithelial cells.

A limitation of our study is that some patients received ophthalmological treatment for their OSD which may have led to less severe OSD. However, patients were divided into having no or mild OSD or moderate‐to‐severe OSD based on the clinical examination which was performed at every visit. Severity was based on the clinical UTOPIA score, and was independent of the use of ophthalmological treatment. For that reason, the ophthalmological treatment did probably not influence our results.

In conclusion, dupilumab‐treated AD patients with moderate‐to‐severe OSD had higher dupilumab tear fluid levels compared to patients with no or mild OSD. This might be explained by a disrupted blood‐tear barrier. Finally, dupilumab binding on conjunctival epithelial cells was found, indicating that dupilumab reaches the ocular surface. Further research is needed to investigate the implications of these increased levels of dupilumab.

## AUTHOR CONTRIBUTIONS

All authors have made substantial contributions to conception and design, or acquisition of data, or analysis and interpretation of data. All authors have been involved in drafting the manuscript or revising it critically and have given final approval of the version to be published.

## CONFLICTS OF INTEREST

Roselie Achten has nothing to disclose. Judith Thijs is a speaker for Sanofi Genzyme and LEO Pharma. Marlot van der Wal has nothing to disclose. Chantal van Luijk is a speaker for Sanofi Genzyme and Santen. Matthijs van Luin has nothing to disclose. Mohsin el Amrani has nothing to disclose. Edward Knol is a speaker and advisory board member for Sanofi Genzyme. Eveline Delemarre has nothing to disclose. Constance den Hartog Jager has nothing to disclose. Marlies de Graaf is a consultant, advisory board member, and/or speaker for AbbVie, Eli Lilly, Leo Pharma, Novartis, Pfizer, Regeneron, and Sanofi‐Genzyme. Daphne Bakker is a speaker for Sanofi Genzyme and LEO Pharma. Joke de Boer has nothing to disclose. Femke van Wijk is a speaker and/or consultant for Janssen, Johnson & Johnson, and Takeda. Marjolein de Bruin‐Weller is a consultant, advisory board member, and/or speaker for AbbVie, Almirall, Aslan, Arena, Eli Lilly, Galderma, Janssen, Leo Pharma, Pfizer, Regeneron, and Sanofi‐Genzyme.

## Supporting information

Supporting Information S1
