# Supplementary material for: High dupilumab levels in tear fluid of atopic dermatitis patients with moderate‐to‐severe ocular surface disease
Source: Clin Transl Allergy. 2023 Jan 16;13(1):e12221. doi: 10.1002/clt2.12221 (PMC9842774; doi:10.1002/clt2.12221)
Supplement: Supplementary file 1 — Supporting Information S1 [file CLT2-13-e12221-s001.docx]

**Supporting information**

**Table S1. Dupilumab levels in tear fluid and serum during dupilumab treatment categorized by OSD severity at the collection moment**

|  | **No or mild OSD at tear fluid collection** | **Moderate-to-severe OSD at tear fluid collection** | **P-value** |
| --- | --- | --- | --- |
| **4 weeks after start dupilumab (n=44**^†^**)** |  |  |  |
| *Tear fluid* |  |  |  |
| Number of patients | 27 | 16 |  |
| Missing | 1 | 0 |  |
| Dupilumab level in tear fluid (mg/L),  median (IQR) | 0.22 (0.13-0.55) | 0.25 (0.19-0.61) | 0.353 |
|  |  |  |  |
| *Serum* |  |  |  |
| Number of patients | 26 | 16 |  |
| Missing | 2 | 0 |  |
| Dupilumab level in serum (mg/L),  median (IQR) | 79.4 (63.2-110.5) | 62.1 (52.0-77.0) | 0.043 |
|  |  |  |  |
| **28 weeks after start dupilumab (n=48)** |  |  |  |
| *Tear fluid* |  |  |  |
| Number of patients | 25 | 19 |  |
| Missing | 2 | 2 |  |
| Dupilumab level in tear fluid (mg/L),  median (IQR) | 0.29 (0.16-0.60) | 0.55 (0.35-1.31) | 0.021 |
|  |  |  |  |
| *Serum* |  |  |  |
| Number of patients | 22 | 19 |  |
| Missing | 5 | 2 |  |
| Dupilumab level in serum (mg/L),  median (IQR) | 105.1 (65.7-129.6) | 100.3 (79.8-117.9) | 0.814 |
|  |  |  |  |
| **Onset or worsening DAOSD (n=23)** |  |  |  |
| *Tear fluid* |  |  |  |
| Number of patients | 3 | 14 |  |
| Missing | 2 | 4 |  |
| Dupilumab level (mg/L), median (IQR) | 0.16 (0.09-0.55) | 1.07 (0.28-2.69) | 0.047 |

Severity of OSD is based on eye with the highest severity within a patient. P-values were calculated with Mann-Whitney U tests. Abbreviations: DAOSD, dupilumab-associated ocular surface disease; IQR, interquartile range; OSD, Ocular surface disease.

^†^ 4 patients did not complete their week 4 visit due to the COVID19 pandemic.

**Table S2. Patient characteristics of patients in which dupilumab binding on conjunctival cells obtained by conjunctival impression cytology was measured.**

|  | **Dupilumab measured in CIC suspensions (n=5)** | **Dupilumab measured in CIC suspensions of AD controls (n=3)** | **IgG4 and IL-4Rα measured in CIC suspensions (n=4)** |
| --- | --- | --- | --- |
| **Age (years), median (IQR)** | 28.0 (22.5 – 48.0) | 39.0 (24.0 – N/A) | 50.5 (27.3 – 64.0) |
| **Men, n (%)** | 2 (40.0) | 2 (66.7) | 3 (75.0) |
| **EASI score baseline, median (IQR)** | 10.5 (1.8 – 19.7) | 14.5 (13.1 – N/A) | 14.8 (7.9 – 21.6) |

Abbreviations: AD, atopic dermatitis; CIC, conjunctival impression cytology; EASI, Eczema


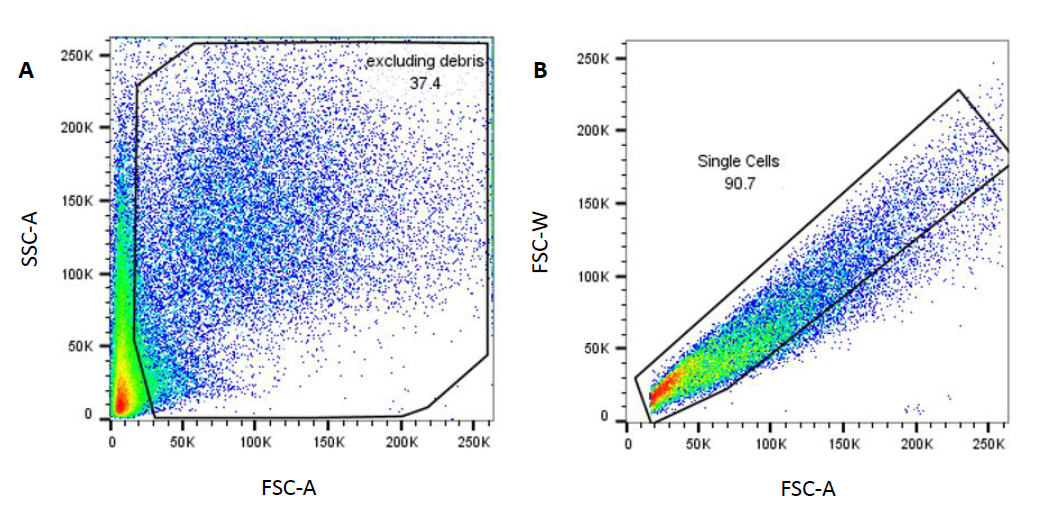


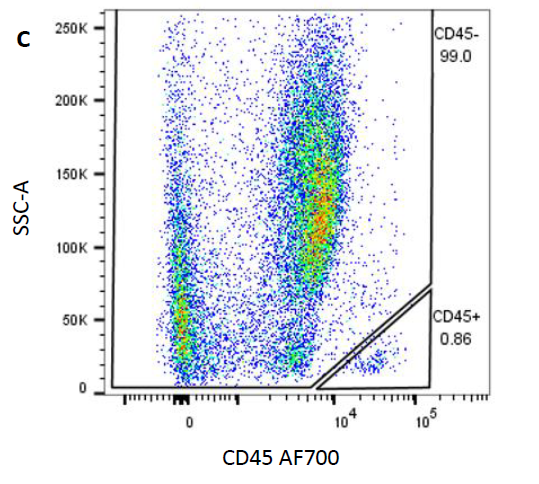


**Figure S1.** **The different steps to gate CD45- cells are depicted.** Debris was excluded (**A**), single cells were gated (**B**) and finally CD45- cells were gated (**C**). Median Fluorescence Intensity (MFI) of IL-4Rα PE and IgG4 biotin – streptavidin APC was determined within CD45- population.
